# Clinical and cardiopulmonary predictors of functional recovery and complications after transcatheter aortic valve implantation: Protocol of a prospective interventional study

**DOI:** 10.1371/journal.pone.0348568

**Published:** 2026-05-15

**Authors:** Samira Martínez-Otero, Marc Giménez-Milà, María José Arguis, Ander Regueiro, Juan José Rodriguez-Arias, María Sanz de la Garza, Alejandro Berenguel, Alejandro Gadella, Laura Fuertes Kenneally, Graciela Martínez-Pallí

**Affiliations:** 1 Department of Anaesthesia and Intensive Care, Hospital Clinic de Barcelona, Barcelona, Spain; 2 Universitat de Barcelona Barcelona, Barcelona, Spain; 3 IDIBAPS, Institut d’Investigació August Pi i Sunyer (IDIBAPS), Barcelona, Spain; 4 Cardiology Department, Hospital Clínic de Barcelona Barcelona, Barcelona, Spain; 5 Cardiology Department, Hospital Universitario de Toledo, Toledo, Spain; 6 Cardiology Department, Hospital General de Alicante, Alicante, Spain; Kurume University School of Medicine, JAPAN

## Abstract

**Introduction:**

Transcatheter Aortic Valve Implantation (TAVI) has emerged as a less invasive alternative to surgical aortic valve replacement, especially for high-risk patients. While TAVI is expected to improve symptoms and functional status, clinical recovery is often heterogeneous, and subjective assessments may not fully capture the degree of improvement. To our knowledge, the changes in functional capacity following TAVI have not been well explored using cardiopulmonary exercise testing (CPET).The study aims to characterise mid-term changes in exercise tolerance after TAVI and identify clinical and functional predictors of improvement in exercise capacity and complications after TAVI.

**Methods and analysis:**

A total of 161 patients with severe aortic stenosis scheduled for TAVI will be prospectively enrolled across three expert centres. Each will undergo clinical assessment and incremental CPET within two weeks before and four to six weeks after the procedure. The primary outcome is a change in VO₂ peak and VO₂ at the anaerobic threshold. Secondary outcomes include exploratory associations between baseline characteristics and observed changes in functional capacity, quality of life and complications.

**Ethics and dissemination:**

The bioethics committee of the Hospital Clínic de Barcelona, Spain, approved this protocol (HCB/2024/0782). All the participating centres obtained local approval prior to patient recruitment. The findings will be published in a peer-reviewed journal and submitted to relevant conferences.

**Trial registration:**

ClinicalTrials.gov NCT06833762 (registered 10th of March 2025).

## Introduction

Aortic stenosis (AS) is the most prevalent primary valvular disease in Europe [1], leading to symptoms such as dyspnoea, angina and syncope and reduced survival when severe [2]. No medical therapy modifies disease progression and valve replacement is the only definitive treatment. Transcatheter Aortic Valve Implantation (TAVI) has emerged as a minimally invasive alternative to surgical aortic valve replacement (SAVR) [3–5] owing to the lower risk of complications [6,7] and non-inferiority compared to SAVR in intermediate and low-risk patients at 1, 2 and 5-year follow-up [8–13].

While TAVI effectively relieves valvular obstruction, clinical recovery remains heterogeneous. Most patients experience symptomatic relief, yet functional improvement varies significantly despite satisfactory procedural and echocardiographic outcomes [14]. Subjective measures such as the New York Heart Association (NYHA) classification are often used, but inter-observer agreement is limited (≈ 55%) [15–17]. Cardiopulmonary exercise testing (CPET) is recognized as the gold standard for quantifying functional capacity by integrating respiratory, cardiovascular, and muscular responses during exercise. It provides objective metrics (such as peak VO₂, VE/VCO₂ slope, and anaerobic threshold) that correlate with clinical outcomes and prognosis in several clinical scenarios [18,19]. Exercise testing has been shown to be safe and well tolerated in patients with aortic stenosis when performed with supervised incremental protocols [20].

Existing CPET studies in aortic stenosis are limited to small, single-centre cohorts in surgical patients [21] or focus on very early post-TAVI changes [22]. These studies often lack comprehensive assessments of frailty, which is a critical determinant of recovery in the typically elderly TAVI population [23]. To date, no multicentre study has characterised early CPET trajectories using a uniform protocol at a more robust follow-up interval.

This prospective, multicenter study addresses these gaps by evaluating CPET changes six weeks after TAVI, integrated with frailty and quality of life assessments. We hypothesise that: (1) the changes in functional capacity after TAVI are objectively measurable by CPET, and (2) that baseline clinical and functional characteristics can predict the degree of improvement in symptoms.

## Methods and analysis

### Study design

Prospective, multicentre, investigator-initiated, single-arm, low-interventional study without randomisation or blinding. CPET and complementary clinical assessments are performed before and six weeks after TAVI, with a six-month follow-up call. The study is classified as low-interventional as it does not alter the clinical management of TAVI; all additional tests (CPET, strength, and frailty) are non-invasive. All clinical management and medical treatments remain at the total discretion of the patient’s attending physicians; study investigators will not perform any modifications to the patient’s pharmacological or therapeutic regimen. In this study a CE (European conformity) marked health product is being used.

### Study setting

Hospital Clínic de Barcelona (coordinating centre), Hospital Universitario de Toledo, and Hospital General de Alicante in Spain.

### Patient and public involvement

Patients were not involved in the study design or protocol development. Results will be shared with participants after publication at their request.

### Eligibility criteria

**Inclusion criteria:**

- Adults (≥ 18 years) with severe aortic stenosis accepted for elective transfemoral TAVI.
- Ability to provide informed consent and perform CPET, which presupposes the absence of musculoskeletal or orthopedic conditions that would preclude completion of a cycle ergometer exercise test

**Exclusion criteria:**

- AS with a valve area ≤ 0.6 cm², mean gradient ≥ 60 mmHg or Peak Velocity > 5 m/s.
- Prior cardiogenic syncope.
- Exercise-induced arrhythmia.
- Dynamic LVOT obstruction (≥ 30 mmHg).
- Concomitant coronary artery disease pending percutaneous coronary intervention.
- Non-elective or valve-in-valve procedures.
- Physical inability to perform exercise testing or inability to consent.

### Outcome measures

**Primary outcomes:**

- To determine the impact of TAVI on mid-term functional capacity in terms of changes in CPET parameters (mainly, VO_2_ peak and %VO_2_ peak predicted, VO_2_ at anaerobic threshold, VE/VCO₂ slope).
- To identify clinical and functional pre-TAVI factors that predict improvement in functional capacity after TAVI.

**Secondary outcomes:**

- To explore associations between baseline CPET parameters and quality-of-life improvement.
- To explore associations between baseline CPET parameters and complications.
- To explore associations between CPET parameters and echocardiographic or frailty measures.

### Study timeline and procedures

**Fig 1:**
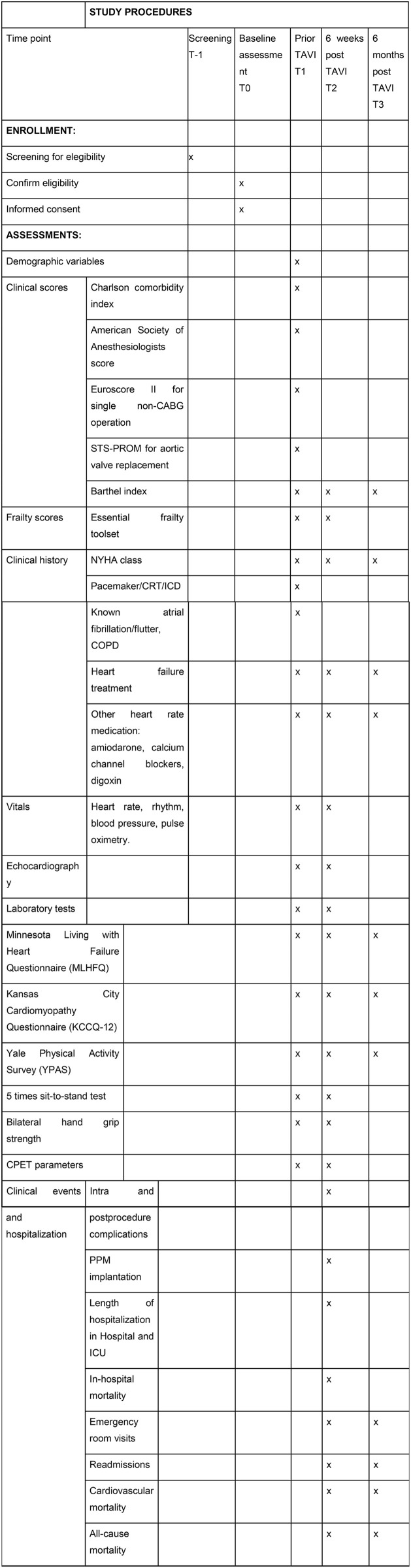
SPIRIT participant timeline, schedule of enrollment and assessment. CABG: coronary artery bypass graft. STS-PROM: Society of Thoracic Surgeons Predicted Risk of Mortality. NYHA: New York Heart Association. CRT: Cardiac Resynchronization Therapy. ICD: Implantable Cardioverter-Defibrillator. COPD: Chronic obstructive pulmonary disease. ACE: Angiotensin-converting enzyme. ARBs: Angiotensin II receptor blockers. SGLT-2i: Sodium-glucose Cotransporter-2 inhibitors. MRA: Mineralocorticoid receptor antagonists. LVEF: Left ventricular ejection fraction. RV: Right ventricle. CPET: Cardiopulmonary exercise testing. NT-pro-BNP: N-terminal pro brain natriuretic peptide. AV: atrioventricular. LBBB: left bundle branch block. AF: atrial fibrillation PPM: permanent pacemaker. AKI: acute kidney injury. ICU: Intensive Care Unit.

At baseline (T1), demographic and clinical data, NYHA class, comorbidity scores (EuroSCORE II, Charlson Index), frailty (Essential Frailty Toolset), physical activity (Yale Physical Activity Survey), functional status (Duke Activity Status Index), cognitive impairment (Mini-Mental State Examination 2) and quality of life (Minnesota Living with Heart Failure Questionnaire) will be recorded (see Fig 1). Laboratory and echocardiographic parameters (including N-terminal pro-B-type natriuretic peptide (NT-pro-BNP), haemoglobin, renal function, Left ventricular ejection fraction (LVEF), mean aortic gradient) will be collected.

Participants will undergo incremental CPET on a cycle ergometer with continuous gas-exchange analysis. The safety of CPET in aortic stenosis, including severe disease, is well established when conducted with appropriate monitoring and ramp protocols, with very low rates of adverse events reported in contemporary studies [20]. All tests in this study will be supervised by trained clinicians and conducted using standardised POETTS-aligned safety procedures. The exercise test will be conducted by a physiotherapist and supervised by a physician using a standard incremental cardiopulmonary exercise testing on cycle ergometer (Ergoline 900, Ergoline, Bitz, Germany (CE-0123) and Ergocard Professional, Medisoft, Sorinnes, Belgium (CE-1434)). Based on the patient’s anticipated exercise capacity, incremental ramp protocols will be applied with gradual increases in pedal resistance, maintaining a pedal cadence higher than 50–60 rpm to ensure a consistent workload. The test will start with a 2-minute warm-up period at free wheel. The protocols are designed to last between 8–12 minutes, ensuring a gradual increase in exertion and preventing sudden spikes in workload that could lead to early fatigue or other complications. Once the patient reaches their maximum effort, the test will stop and they will be allowed to cool down for 2 minutes. Variables will include VO₂ at anaerobic threshold and peak, oxygen pulse, respiratory exchange ratio, VE/VCO₂ slope, oxygen-uptake efficiency slope and heart rate at AT. Based on previous studies [24], a ΔVO₂) > 2.5 ml/kg/min or >10% increase in VO_2_ max between pre- and post-TAVI tests will denote a positive functional response.

Hand-grip strength and five-times-sit-to-stand tests will evaluate muscular strength. These functional assessments, along with the Essential Frailty Toolset (EFT), are specifically prioritized to provide a bedside-accessible evaluation of peripheral muscle function and physical frailty, which are known determinants of cardiopulmonary exercise performance in the TAVI population.

At six weeks (T2), all baseline assessments, including a record of cardiovascular medications, will be repeated. Clinical complications, such as vascular or bleeding events, conduction disturbances, arrhythmias, stroke, renal injury, or mortality, etc., hospital stay, readmission and survival will be recorded. The 6-week reassessment corresponds to the routine post-TAVI clinical follow-up and is intended to characterise the early phase of functional recovery after relief of valvular obstruction. While longer-term physiological adaptations may continue beyond this timeframe, the study focuses on the early trajectory to minimise loss to follow-up and capture the immediate effects of afterload reduction.

**Fig 2:**
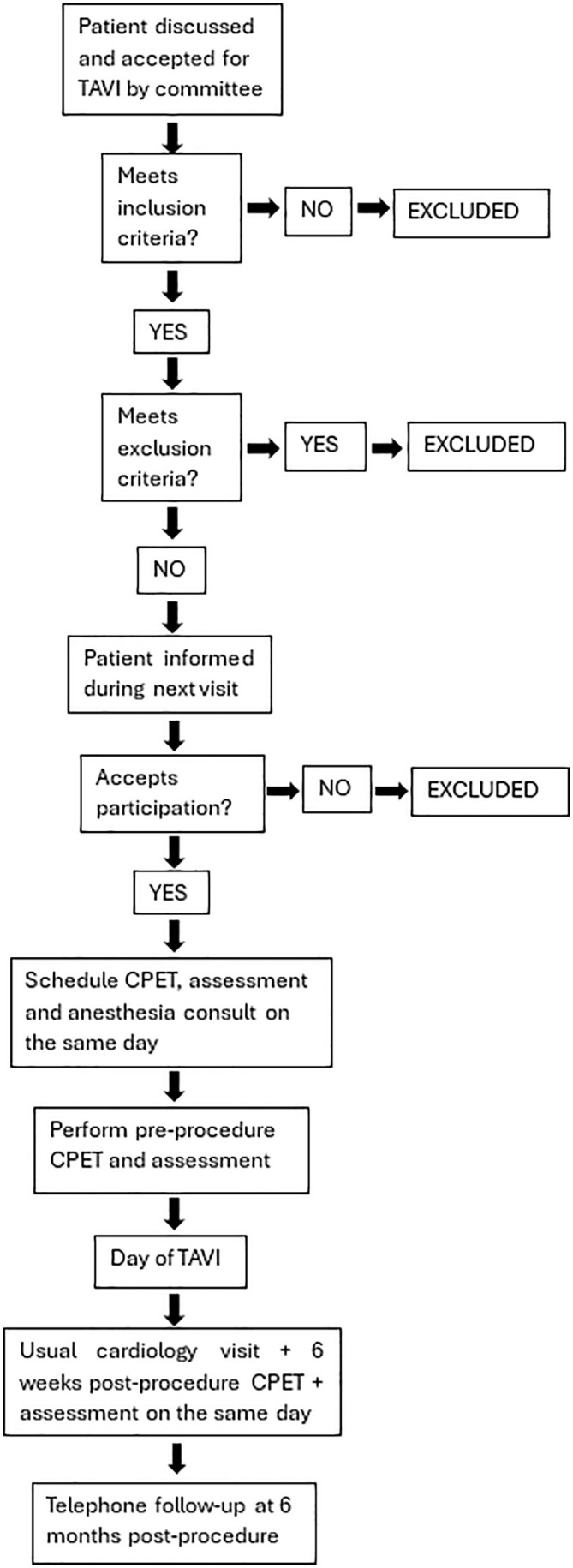
Flowchart of recruitment and assessments during study.

At six months post-procedure, patients will be contacted by telephone to assess survival status, record any interim clinical events, symptoms or changes to medical treatment, and repeat the quality-of-life and physical-activity questionnaires (Fig 2.). It should be noted that the participating centers do not currently offer a standard cardiac rehabilitation program for this patient population.

Participant recruitment is estimated to be completed by the end of 2026, data collection by beginning of 2027 and results expected to be disseminated by mid 2027.

### Sample size

Previous studies evaluating exercise testing in patients undergoing percutaneous structural heart interventions have included small cohorts of 11–30 patients [23,25]. Based on recent data from Bellander et al. [21], approximately 24% of patients undergoing aortic valve replacement were classified as responders according to objective CPET criteria. Therefore, the sample size for this study was calculated assuming a 24% response rate in the reference population, aiming to detect a 10% difference in response with 80% statistical power and a two-tailed significance level of 5%. Based on these assumptions, the required sample size was estimated at 153 patients. Considering an anticipated dropout rate of 5%, the final sample size was set at 161 participants. No interim analysis was planned.

### Methods against bias

The prospective design reduces historical bias. This study will collect patients in three different centres to increase generalisability. The same CPET equipment and cycle ergometer will be used in all three centres to reduce equipment variability. Sex and gender differences will be analysed in recruitment and outcome interpretation. Data collection tools will capture gender identity and expression in a respectful and inclusive way such as “Male,” “Female,” “Non-binary,” “Prefer not to disclose,” or free-text fields for self-identification.

### Statistical analysis

Categorical data will be summarised as n (%); continuous data as mean ± SD or median (IQR). Normality of distribution will be tested using the Shapiro-Wilk test. Fisher’s exact, McNemar, T-Student’s, Mann-Whitney U or Friedman tests will be used as appropriate. Exploratory linear regression models will assess associations between a limited, set of baseline variables and continuous ΔCPET values. Additional variables (e.g., QoL scores, strength tests, comorbidities) will be examined in univariate analyses.

Penalised regression methods may be used where appropriate.

All such analyses will be interpreted as exploratory. Complications and QoL changes will be summarised descriptively, and any associations with baseline CPET parameters will be treated as exploratory. Furthermore, a prespecified subgroup analysis will be performed based on baseline symptom status (NYHA class I vs. NYHA class II-IV) to assess if the magnitude of functional recovery differs between symptomatic and asymptomatic patients.

### Data handling

Data will be stored in coded form on institutional REDCap servers for 5 years after study completion (REDCap Clínic). Only authorised investigators will access identifiable data; all analyses will use de-identified datasets. No biological samples will be collected.

Patient confidentiality will be ensured by assigning a study code to each of the participants. The association of patient identification and code will be stored on a separate database on the Hospital Clinic servers. Only the principal investigator will have access to the encoding database.

### Ethics statement

The study complies with the Declaration of Helsinki (2013) and EU Regulations 2016/679 and 2017/745. Approval was granted by the Hospital Clínic de Barcelona Research Ethics Committee – (HCB/2024/0782 August 2024) being chaired by Prof Josep Maria Miró Meda. All participating centres obtained local approval prior to patient recruitment which started in 2025.

All participants will be adequately informed verbally and through a written patient information form. Participants will sign informed consent before inclusion and may withdraw at any time. Any adverse event during testing will be adequately reported Eventual protocol modifications will be notified to Research Ethics Commitee.

All the participating centres obtained local approval prior to patient recruitment. The findings will be published in a peer-reviewed journal and submitted to relevant conferences.

Data from this research will be made available to the scientific community in a timely manner regardless of the results. The investigators will comply with internationally agreed requirements for authorship and will approve any manuscripts prior to submission.

### Strengths and limitations of this study

- To our knowledge, this is the first prospective multicentre study to objectively evaluate the functional changes after TAVI using cardiopulmonary exercise testing.
- It integrates objective physiological metrics, frailty indices and quality-of-life questionnaires, offering a comprehensive evaluation of recovery after TAVI.
- The absence of a control group may limit causal inference between TAVI and functional improvement.
- The relatively small sample size may limit external validity.
- The lack of objective quantification of muscle mass through advanced imaging (e.g., CT-based metrics) is acknowledged as a potential limitation of the current study design.
- Patients unable to complete testing may represent a frailer subgroup, introducing a potential selection bias.

## Supporting information

S1 File Data. (PDF)

S2 File SPIRIT 2025 editable checklist. (DOC)
