# Supplementary material for: Clinical and cardiopulmonary predictors of functional recovery and complications after transcatheter aortic valve implantation: Protocol of a prospective interventional study
Source: PLoS One. 2026 May 15;21(5):e0348568. doi: 10.1371/journal.pone.0348568 (PMC13178984; doi:10.1371/journal.pone.0348568)
Supplement: S1 File — (PDF) [file pone.0348568.s001.pdf]

1. General information

1.1. Study identification:

- Title: **Determining clinical and CPET parameters that predict improvement in functional capacity and complications after transcatheter aortic valve implantation**
- Protocol ID code or number: TAVI-CPET
- Version and date: 2 21/08/2024

1.2. Promoter ID

- Name and address.

1.3 Principal investigators in our center

Dr. Samira Martínez Otero (SMO)

Direction: Graciela Martínez Pallí, Marc Giménez Milà

Collaborating investigators: Dr. María José Arguis Giménez, Dr. Juan José Rodríguez Arias, Dr. Ander Regueiro, physiotherapist.

1.4 Principal investigators in participating centers

Hospital Universitario de Toledo: Dr. Alejandro Berenguel

Hospital General de Alicante: Dr. Balmis, Dr. Laura Fuertes Kenneally

2. Justification

Aortic stenosis (AS) is characterized by a fixed obstruction to left ventricular outflow and it is the most common primary valve disease in Europe[1]. When this obstruction becomes severe it significantly decreases patient survival and can cause symptoms such as dyspnea, chest pain and syncope[2]. No medical therapies have been shown to change disease progression, therefore, in this scenario aortic valve replacement is indicated.

In the last decade, studies show a significant rise in Transcatheter Aortic Valve Implantation (TAVI) procedures compared to traditional surgical aortic valve replacement (SAVR)[3]. This trend is reflected in the European Society of Cardiology[4] and the American College of Cardiology Guidelines[5] for the management of patients with valvular heart disease and is likely due to the minimally invasive nature and potential benefits of TAVI, particularly for high-risk patients. Studies suggest TAVI may be associated with a lower risk of certain complications like new-onset atrial fibrillation, major bleeding and acute kidney injury as compared to SAVR[6]. However, despite its good safety profile, TAVI is not without risks. Some of the common complications are heart block requiring pacemaker implantation, vascular and bleeding complications, stroke, acute kidney

injury and new atrial fibrillation. Rare complications include aortic dissection, ventricular perforation, annular rupture, mitral valve disruption and coronary obstruction[7].

Nonetheless, in recent years, several trials have shown non-inferiority of TAVI vs SAVR in intermediate and low-risk patients at 1, 2 and 5-year follow-up (NOTION[8,9], PARTNER 2[10], PARTNER 3[11], SURTAVI[12], Evolut Low Risk[13]). This is creating a move towards TAVI becoming the strategy of choice in a larger group of patients.

After a successful TAVI procedure, the expected clinical result is a reduction in symptoms, noticeable improvement in exercise tolerance (i.e., functional or aerobic capacity), and improvement in quality of life. However, in our experience during clinical follow-up this is not always the case[14], or is it difficult to determine the degree of improvement. In some patients, symptoms do not improve as we would expect despite a successful procedure with good echocardiographic results.

To our knowledge, the potential functional benefit of TAVI has not been well explored using cardiopulmonary exercise testing (CPET), a method considered the gold standard for measuring functional capacity. A significant increase in exercise time, oxygen consumption and ventilatory anaerobic threshold (by CPET) has been shown in a small sample of 11 patients with heart failure, 6 months after percutaneous mitral valve repair[15]. Another study including both patients who underwent SAVR and TAVI, did not observe an improvement in VO_2 max after 9 months of the procedure. A preoperative transvalvular gradient < 40mmHg, high preoperative Brain Natriuretic Peptide, previous beta-blocker treatment and patients with a pacemaker were associated with a lower relative change in VO_2 max[16]. A recent small study published in 2020 by a group from Switzerland, explored the short-term changes in functional capacity in 30 patients after TAVI. They did not use CPET but electrical cardiometry and near-infrared spectroscopy and they showed improved median workload, cardiac index and cerebral and muscle tissue oximetry at 5 days compared to baseline[17]. No data were reported about the potential improvement in aerobic capacity.

On the other hand, most patients who undergo TAVI are elderly, and characterized by a high frailty degree, multiple comorbidities, and muscular deconditioning, which involves not only sarcopenia but also mitochondrial dysfunction, all well described in the heart failure population[18]. Our premise is that there are multiple factors that contribute to their limitation in functional capacity beyond the aortic stenosis itself. This makes it challenging to determine which patients will truly benefit from TAVI and those whose symptoms will partially or not improve despite eliminating the obstruction.

Surgical risk scores such as the European System for Cardiac Operative Risk Evaluation II (EuroSCORE II) and the Society of Thoracic Surgeons (STS) predicted risk of mortality (PROM) score have been validated for surgical interventions. However, they do not contemplate frailty and other comorbidities that impact outcomes in transcatheter procedures[4]. Hence, new scores have been specifically developed to estimate the risk in patients undergoing TAVI (PARTNER, FRANCE 2), although there is no standardized method to evaluate the potential benefit of the procedure. Furthermore, clinical improvement is mostly assessed by the patient's self-reported

change in symptoms, and the clinician's assignment of a New York Heart Association (NYHA) class based on this information. However, it is well established that interobserver agreement of NYHA classification is only around 55%, and there is no consistent method to assign a NYHA class[19–21]. Therefore, clinical improvement is still essentially a subjective measurement.

In this scenario, CPET, a well-established tool in the preoperative risk assessment of surgical patients[22], could be a promising tool not only to objectively determine the degree of improvement in functional capacity after TAVI but also to predict the clinical response to TAVI. CPET has been shown to be useful in determining exercise-limiting factors and pathophysiologic mechanisms, and assessing the contribution of cardiovascular, pulmonary and muscular etiology in coexisting disease[23].

We believe the utility of performing CPET, along with strength tests and frailty scores, in these patients is potentially two-fold. Firstly, identifying which patients have an objective improvement in functional capacity, and have therefore truly benefited from TAVI. Secondly, predicting which group of patients will show this improvement. This could open a door to implementing these strategies in a systematic way to aid in future decisions to proceed with TAVI or not. Identifying patients who are unlikely to benefit from TAVI in their current state could prompt us to offer alternatives that target their specific limitations and needs.

The present study aims to: 1) characterize the mid-term exercise tolerance response to TAVI, and 2) determine the clinical and functional factors that determine the magnitude of this response.

According to the Perioperative Exercise Testing and Training Society (POETTS) guidelines[22], adequate conditions for performing perioperative CPET are considered to be: adequate hemodynamic monitoring and respired gas analysis, cycle ergometry preferred over ramp test, equipment that must be routinely calibrated, a minimum of 2 members of staff must be available, one should be an advanced CPET practitioner and at least one should have life support competencies; a physician, a resuscitation team and equipment must be available, a standardized protocol should be used. All these conditions are met in our center.

2.1 Bibliography

1. Iung B, Delgado V, Rosenhek R, et al. Contemporary Presentation and Management of Valvular Heart Disease: The EURObservational Research Programme Valvular Heart Disease II Survey. *Circulation*. 2019;140(14):1156-1169. doi:10.1161/CIRCULATIONAHA.119.041080
2. Kvaslerud AB, Santic K, Hussain AI, et al. Outcomes in asymptomatic, severe aortic stenosis. *PLoS One*. 2021;16(4):e0249610. doi:10.1371/journal.pone.0249610
3. Lee G, Chikwe J, Milojevic M, et al. ESC/EACTS vs. ACC/AHA guidelines for the management of severe aortic stenosis. *Eur Heart J*. 2023;44(10):796-812. doi:10.1093/eurheartj/ehac803
4. Vahanian A, Beyersdorf F, Praz F, et al. 2021 ESC/EACTS Guidelines for the management of valvular heart disease. *Eur Heart J*. 2022;43(7):561-632. doi:10.1093/eurheartj/ehab395
5. Writing Committee Members, Otto CM, Nishimura RA, et al. 2020 ACC/AHA Guideline for the Management of Patients With Valvular Heart Disease: A Report of the American College of Cardiology/American Heart Association Joint Committee on Clinical Practice Guidelines. *J Am Coll Cardiol*. 2021;77(4):e25-e197. doi:10.1016/j.jacc.2020.11.018
6. Swift SL, Puehler T, Misso K, et al. Transcatheter aortic valve implantation versus surgical aortic valve replacement in patients with severe aortic stenosis: a systematic review and meta-analysis. *BMJ Open*. 2021;11(12):e054222. doi:10.1136/bmjopen-2021-054222
7. Dalby M, Panoulas V. Transcatheter aortic valve implantation: complications. UptoDate.
8. Thyregod HGH, Jørgensen TH, Ihlemann N, et al. Transcatheter or surgical aortic valve implantation: 10-year outcomes of the NOTION trial. *Eur Heart J*. 2024;45(13):1116-1124. doi:10.1093/eurheartj/ehae043
9. Thyregod HGH, Steinbrüchel DA, Ihlemann N, et al. Transcatheter Versus Surgical Aortic Valve Replacement in Patients With Severe Aortic Valve Stenosis. *J Am Coll Cardiol*. 2015;65(20):2184-2194. doi:10.1016/j.jacc.2015.03.014
10. Leon MB, Smith CR, Mack MJ, et al. Transcatheter or Surgical Aortic-Valve Replacement in Intermediate-Risk Patients. *N Engl J Med*. 2016;374(17):1609-1620. doi:10.1056/NEJMoa1514616
11. Shahim B, Malaisrie SC, George I, et al. Postoperative Atrial Fibrillation or Flutter Following Transcatheter or Surgical Aortic Valve Replacement: PARTNER 3 Trial. *JACC Cardiovasc Interv*. 2021;14(14):1565-1574. doi:10.1016/j.jcin.2021.05.026
12. Van Mieghem NM, Deeb GM, Søndergaard L, et al. Self-expanding Transcatheter vs Surgical Aortic Valve Replacement in Intermediate-Risk Patients: 5-Year Outcomes of the SURTAVI Randomized Clinical Trial. *JAMA Cardiol*. 2022;7(10):1000-1008. doi:10.1001/jamacardio.2022.2695
13. Popma JJ, Deeb GM, Yakubov SJ, et al. Transcatheter Aortic-Valve Replacement with a Self-Expanding Valve in Low-Risk Patients. *N Engl J Med*. 2019;380(18):1706-1715. doi:10.1056/NEJMoa1816885
14. Nuche J, Ternacle J, Avvedimento M, et al. Incidence, predictors, and prognostic significance of impaired functional status early after transcatheter aortic valve replacement. *Rev Esp Cardiol (Engl Ed)*. 2024;77(5):396-407. doi:10.1016/j.rec.2023.11.003

15. Benito-González T, Estévez-Loureiro R, Garrote-Coloma C, et al. MitraClip improves cardiopulmonary exercise test in patients with systolic heart failure and functional mitral regurgitation. *ESC Heart Fail*. 2019;6(4):867-873. doi:10.1002/ehf2.12457
16. Le VDT, Jensen GVH, Kjøller-Hansen L. Observed change in peak oxygen consumption after aortic valve replacement and its predictors. *Open Heart*. 2016;3(1):e000309. doi:10.1136/openhrt-2015-000309
17. Suppan M, Barcelos G, Luise S, et al. Improved Exercise Tolerance, Oxygen Delivery, and Oxygen Utilization After Transcatheter Aortic Valve Implantation for Severe Aortic Stenosis. *CJC Open*. 2020;2(6):490-496. doi:10.1016/j.cjco.2020.06.005
18. Pedriali G, Morciano G, Patergnani S, et al. Aortic Valve Stenosis and Mitochondrial Dysfunctions: Clinical and Molecular Perspectives. *Int J Mol Sci*. 2020;21(14). doi:10.3390/ijms21144899
19. Goldman L, Hashimoto B, Cook EF, Loscalzo A. Comparative reproducibility and validity of systems for assessing cardiovascular functional class: advantages of a new specific activity scale. *Circulation*. 1981;64(6):1227-1234. doi:10.1161/01.cir.64.6.1227
20. Raphael C, Briscoe C, Davies J, et al. Limitations of the New York Heart Association functional classification system and self-reported walking distances in chronic heart failure. *Heart*. 2007;93(4):476-482. doi:10.1136/hrt.2006.089656
21. Bennett JA, Riegel B, Bittner V, Nichols J. Validity and reliability of the NYHA classes for measuring research outcomes in patients with cardiac disease. *Heart Lung*. 2002;31(4):262-270. doi:10.1067/mhl.2002.124554
22. Levett DZH, Jack S, Swart M, et al. Perioperative cardiopulmonary exercise testing (CPET): consensus clinical guidelines on indications, organization, conduct, and physiological interpretation. *Br J Anaesth*. 2018;120(3):484-500. doi:10.1016/j.bja.2017.10.020
23. American Thoracic Society, American College of Chest Physicians. ATS/ACCP Statement on cardiopulmonary exercise testing. *Am J Respir Crit Care Med*. 2003;167(2):211-277. doi:10.1164/rccm.167.2.211
24. Mueller S, Winzer EB, Duvinage A, et al. Effect of High-Intensity Interval Training, Moderate Continuous Training, or Guideline-Based Physical Activity Advice on Peak Oxygen Consumption in Patients With Heart Failure With Preserved Ejection Fraction: A Randomized Clinical Trial. *JAMA*. 2021;325(6):542-551. doi:10.1001/jama.2020.26812

3. Hypothesis

1. The potential benefits of TAVI on functional capacity are objectively measurable by CPET.
2. Patients with AS have different degrees of functional response to TAVI. There are clinical and functional factors prior to TAVI that can predict the degree of response.

4. Objectives and endpoints

Primary outcome:

To determine the impact of TAVI on mid-term functional capacity in terms of change in CPET parameters: VO_2 max, VO_2 at anaerobic threshold.

Secondary outcomes:

- To identify clinical and functional pre-TAVI factors that predict improvement in functional capacity after TAVI (i.e., VO_2 max increase > 2.5 ml/kg/min).
- To identify clinical, echocardiographic and functional pre-TAVI variables that predict improvement in quality of life.
- Relationship between pre-TAVI CPET parameters and in-hospital complications.
- Relationship between pre-TAVI CPET parameters and complications at 6 weeks.
- Relationship between echocardiographic and CPET parameters after TAVI.
- Relationship between frailty and post-TAVI clinical outcomes.

5. Study design

This is a prospective, multi-center study where we will perform a CPET in patients with severe aortic stenosis before and six weeks after TAVI. There will be no control group, randomization or blinding. This study does not have any intervention other than performing a CPET, frailty assessment and strength tests. In this study we will use a CE (European conformity) marked health product.

6. Patient selection

Patients 18 years of age or older, with severe, non-critical, aortic stenosis who are scheduled for transfemoral TAVI in Hospital Clinic de Barcelona and other participating centers and consent to participate in the study.

6.1 Inclusion criteria

All those patients ≥ 18 years of age with severe aortic stenosis approved for transfemoral TAVI who consent to participate in the study.

6.2 Exclusion criteria

- Very severe aortic stenosis, defined as a valve area of ≤0.6 cm^2, mean gradient ≥60 mmHg or Vmax >5 m/s
- Previous cardiogenic syncope
- Proven exercise-induced arrhythmias
- Previously known dynamic left ventricular outflow tract (LVOT) obstruction, defined as LVOT gradient of ≥ 30 mmHg by echocardiography
- Concomitant coronary artery disease pending percutaneous coronary intervention
- Inability to consent
- Physical limitation to perform an exercise test
- Non-elective procedure
- Valve-in-valve procedure

7. Study timeline

Screening and recruitment (T0)

Patients who meet inclusion criteria, who have been discussed in our TAVI committee and are accepted for the procedure, will be eligible for the study. At the time of their next visit with the cardiology team they will be approached by research staff and informed of the study. If they consent, and do not meet any exclusion criteria, they will be scheduled for the baseline CPET, assessment and preanesthetic consult on the same day, in the month leading up to the TAVI.

Baseline assessment (T1)

At this time, we will collect demographic variables, clinical history and vitals (see Table 1), and we will perform the preprocedure exercise test. We will perform spirometry prior to the exercise test. The exercise test will be conducted by a physiotherapist and supervised by a physician using a standard incremental cardiopulmonary exercise testing on cycle ergometer (Ergoline 900, Ergoline, Bitz, Germany (CE-0123) and Ergocard Professional, Medisoft, Sorinnes, Belgium (CE-1434)). Based on the patient's anticipated exercise capacity, incremental ramp protocols will be applied with gradual increases in pedal resistance every 3 minutes, maintaining a pedal cadence higher than 50-60 rpm to ensure a consistent workload. The protocols are designed to last between 8 to 12 minutes, ensuring a gradual increase in exertion and preventing sudden spikes in workload that could lead to early fatigue or other complications. The following variables will be obtained for the analysis: oxygen consumption (VO_2) at anaerobic threshold and peak, oxygen pulse (VO_2 divided by heart rate), respiratory exchange ratio/coefficient (RER) (ratio of VCO_2 to VO_2), VE/VCO_2 slope (ventilatory efficiency, as measured by minute ventilation divided by carbon dioxide production), oxygen uptake efficiency slope, and heart rate at anaerobic threshold (HR at AT).

Based on previous studies, a change in VO_2 peak equal to or greater than 2.5 mL/kg/min between pre and post TAVI will be assumed as a positive response to TAVI in terms of improvement in functional capacity[24].

Participants will also undergo hand grip strength test (Jamar Hydraulic Hand Dynamometer; Sammons Preston, Bolingbrook, Illinois, USA), and 5 times sit-to-stand test. American Society of Anesthesiologists status, Charlson Comorbidity Index, Barthel Index, STS-PROM score and Euroscore II data will be recorded. Physical activity will be measured by the Yale Physical Activity Survey (YPAS), functional capacity by Duke Activity Status Index questionnaire, frailty by the Essential Frailty Toolset. Quality of life will be measured by Minnesota Living with Heart Failure Questionnaire and Kansas City Cardiomyopathy Questionnaire.

Hemoglobin level, albumin, prealbumin, creatinine, glomerular filtration rate and NT-pro-BNP, usually determined before the procedure, will be recorded, as will relevant preprocedure echocardiography parameters (see Table 1).

TAVI will be performed by the regular Cardiology team and in the usual timeframe, in other words, study participation will have no impact on wait times for TAVI, and no clinical decisions will be made based on the results of our assessment.

Postprocedure assessment (T2-3)

Patients will be reassessed using the same tests 6 weeks after the procedure, on the same day as the routine cardiology follow-up visit.

At this time we will also record clinical data such as intra and postprocedure complications: vascular access and bleeding complications, stroke, AV block, LBBB, new AF, aortic dissection, annular rupture, ventricular perforation, mitral valve disruption, coronary obstruction, need for sternotomy, AKI; hospital and intensive care unit (ICU) stay, readmissions, mortality (see Table 1).

The duration of these visits will be approximately 2 hours. Patients will need to avoid caffeine, alcohol, cigarettes, non-prescription drugs and intense exercise on the day of testing. Patients should avoid eating and should drink only water 2 hours prior to the test.

Six months after the procedure we will contact patients by telephone to record symptoms, clinical events and survival at that time point, as well as repeat quality of life and physical activity questionnaires. Patient information forms will be handed out to patients at the first visit, or whenever requested. Signed consent forms will be required before enrollment in the study.

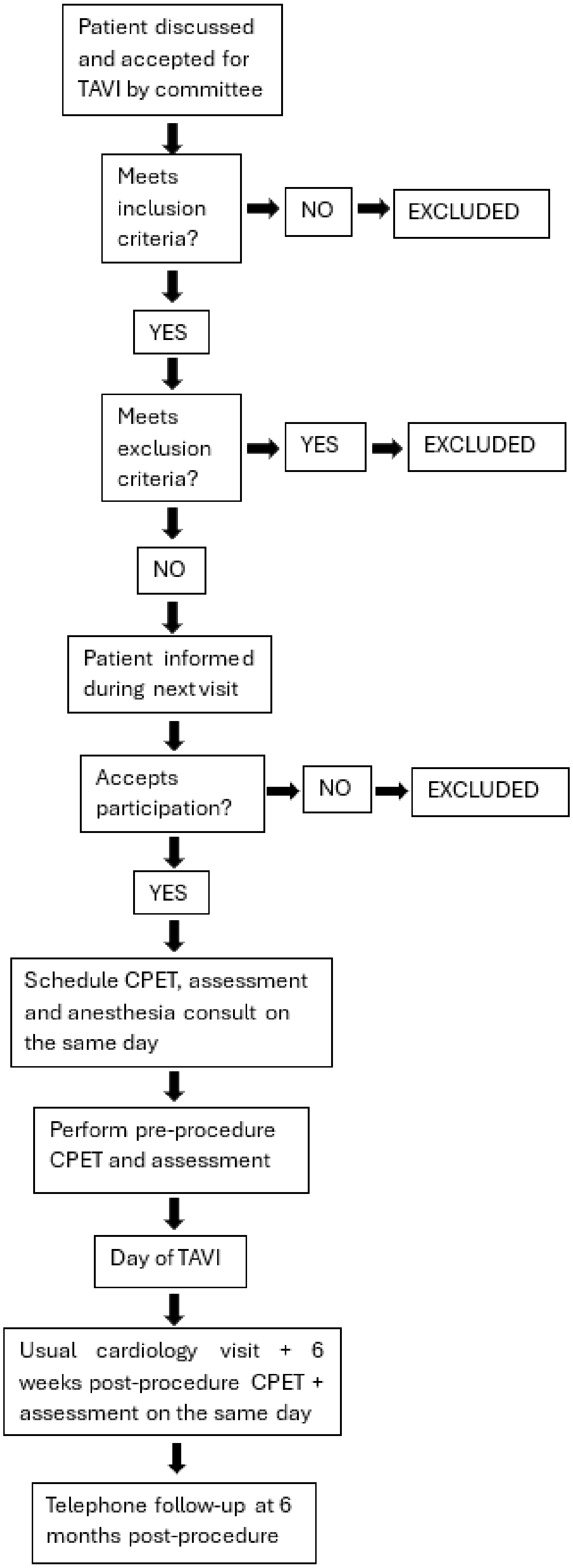
Patient discussed and accepted for TAVI by committee
Meets inclusion criteria?
NO
EXCLUDED
YES
Meets exclusion criteria?
YES
EXCLUDED
NO
Patient informed during next visit
Accepts participation?
NO
EXCLUDED
YES
Schedule CPET, assessment and anesthesia consult on the same day
Perform pre-procedure CPET and assessment
Day of TAVI
Usual cardiology visit + 6 weeks post-procedure CPET + assessment on the same day
Telephone follow-up at 6 months post-procedure

Study procedures and timeline					
		T0<br>Cardiology visit	T1<br>preTAVI	T2<br>6 weeks post TAVI	T3<br>6 months post TAVI
Confirm eligibility		x			
Informed consent		x			
Demographic variables: age, sex, BMI			x		
Clinical scores	Charlson comorbidity index		x		
	American Society of Anesthesiologists score		x		
	Euroscore II for single non-CABG operation		x		
	STS-PROM for aortic valve replacement		x		
	Barthel index		x	x	x
Frailty scores	Essential frailty toolset		x	x	
Clinical history	NYHA class		x	x	x
	Pacemaker/CRT/ICD		x		
	Known atrial fibrillation/flutter		x		
	COPD		x		
	Diuretic treatment		x	x	x
	Heart failure treatment: ACE inhibitors, ARBs, sacubitril-valsartan, SGLT-2i, beta blockers, MRA, ivabradine		x	x	x
	Other heart rate medication: amiodarone, calcium channel blockers, digoxin		x	x	x
Vitals	Heart rate, rhythm, blood pressure, pulse oximetry.		x	x	
Echocar-diography	Mean aortic gradient, degree of aortic insufficiency, LVEF, RV function, degree of mitral regurgitation		x	x	
Bloodwork	Prealbumin, albumin, hemoglobin,		x	x	

	hematocrit, ferritin, transferrin saturation, NT-pro-BNP, creatinine, glomerular filtration rate, lactate				
Minnesota Living with Heart Failure Questionnaire (MLHFQ)			x	x	x
Kansas City Cardiomyopathy Questionnaire (KCCQ-12)			x	x	x
Yale Physical Activity Survey (YPAS)			x	x	x
5 times sit-to-stand test			x	x	
Bilateral hand grip strength			x	x	
CPET parameters			x	x	
Clinical events and hospitalization	Intra and postprocedure complications: vascular access and bleeding complications, stroke, AV block, LBBB, new AF, aortic dissection, annular rupture, ventricular perforation, mitral valve disruption, coronary obstruction, need for sternotomy, AKI.			x	
	PPM implantation			x	
	Length of hospitalization			x	
	ICU/stepdown stay			x	
	In-hospital mortality			x	
	Emergency room visits			x	x
	Readmissions			x	x
	Cardiovascular mortality			x	x
	All-cause mortality			x	x

***Table 1**: CABG: coronary artery bypass graft. STS-PROM: Society of Thoracic Surgeons Predicted Risk of Mortality. NYHA: New York Heart Association. CRT: Cardiac Resynchronization Therapy. ICD: Implantable Cardioverter-Defibrillator. COPD: Chronic obstructive pulmonary disease. ACE: Angiotensin-converting enzyme. ARBs: Angiotensin II receptor blockers. SGLT-2i: Sodium-glucose Cotransporter-2 inhibitors. MRA: Mineralocorticoid receptor antagonists. LVEF: Left ventricular ejection fraction. RV: Right ventricle. CPET: Cardiopulmonary exercise testing. NT-pro-BNP: N-terminal pro brain natriuretic peptide. AV: atrioventricular. LBBB: left bundle branch block. AF: atrial fibrillation PPM: permanent pacemaker. AKI: acute kidney injury. ICU: Intensive Care Unit.*

8. Statistics

8.1 Sample size

Previous similar studies analyzing exercise testing in patients undergoing percutaneous structural heart interventions have included populations of 11 to 30 patients[15,17]. Based on our current volume of TAVI patients, we estimate we will include one patient per week in each of the study centers, with a final population of 120 patients in the study.

8.2 Statistical analysis

Categorical data will be expressed as frequency (%). Quantitative variables will be expressed as mean +- standard deviation or median and interquartile range (IQR) depending on their distribution. Categorical variables will be analyzed with Fisher's exact test, in case of repeated measures McNemar test will be applied. Continuous variables will be compared with Student's t-test for independent groups and Mann-Whitney U test according to each variable distribution, in case of repeated measures Friedman test will be applied.

For the primary outcome, we will utilize a multiple linear regression analysis to establish the relationship between baseline characteristics and changes in continuous CPET variables following TAVI. To assess the relationship between these baseline characteristics and CPET values and the qualitative secondary outcomes, we will employ a multiple logistic regression analysis. For the secondary outcome, defined as a composite of death, heart failure readmission, and in-hospital complications, we will use a Cox proportional hazards regression model. A p-value of 0.05 will be considered for statistical significance.

9. Ethics and legal aspects

The study will be conducted in compliance with the protocol and principles laid down in the latest amendment of the Declaration of Helsinki (Fortaleza, Brazil, October 2013), and in compliance with the Regulation (EU) 2017/745 of the European Parliament and of the Council of 5 April 2017 on medical devices.

All participants will be adequately informed verbally and through a written patient information form, and all questions will be answered. We will request the signature of a consent form prior to enrollment in the study. Patients will be able to withdraw their consent at any given time without any explanation. Any adverse event during testing will be adequately reported.

10. Data handling

We will store encoded data on a RedCap database stored on the Hospital Clinic servers (RedCap Clinic). All collaborating investigators will have access to the coded RedCap database. The data will be stored for 5 years after the completion of the study.

Patient confidentiality will be ensured by assigning a study code to each of the participants. The association of patient identification and code will be stored on a separate database on the Hospital Clinic servers. Only the principal investigator will have access to the encoding database.

11. Data Processing and Records Filing. Data confidentiality

The processing, communication and transfer of personal data of all participants will comply with EU Regulation 2016/679 of the European Parliament and of the Council of April 27, 2016 regarding the protection of natural persons regarding to the processing of personal data and the free circulation of data, and to Organic Law 3/2018, of December 5, on the Protection of Personal Data and guarantee of digital rights. The legal basis that justifies the processing of your data is the consent you give in this act, in accordance with the provisions of article 9 of EU Regulation 2016/679.

The data collected for these studies will be identified only by a code, so no information that would identify the participants will be included. Only the study doctor and his collaborators with the right of access to the source data (medical history) will be able to relate the data collected in the study with the patient's medical history.

The identity of the participants will not be accessible to any other person except in a medical emergency or legal requirement.

Health authorities, the Research Ethics Committee and personnel authorized by the study promoter may have access to identified personal information, when necessary to verify data and procedures of the study, but always maintaining confidentiality in accordance with current legislation.

Only the encrypted data will be transferred to third parties and other countries, which in no case will contain information that can identify the participant directly (such as name and surname, initials, address, social security number, etc.). In the event that this transfer occurs, it would be for the same purpose of the study described and guaranteeing confidentiality.

If a transfer of encrypted data is carried out outside the EU, either in entities related to the hospital center where the patient participates, to service providers or to researchers who collaborate with us, the participants' data will be protected by safeguards such as contracts or other mechanisms established by data protection authorities.

As promoters of the project, we undertake to process the data in accordance with EU Regulation 2016/679 and, therefore, to maintain a record of the processing activities that we carry out and to carry out a risk assessment of the treatments we perform to know what measures we will have to apply and how.

In addition to the rights already contemplated by the previous legislation (access, modification, opposition and cancellation of data, deletion in the new Regulation) participants can now also limit the processing of data collected for the project that is incorrect, request a copy or transfer to a

third party (portability). To exercise these rights, they must contact the principal investigator of the study or the Data Protection Delegate of the Hospital Clinic of Barcelona through protecciodades@clinic.cat. They also have the right to contact the Data Protection Agency if they are not satisfied. to.

Data cannot be deleted even if a patient leaves the study, to ensure the validity of the research and to comply with legal duties and drug authorization requirements.

The Researcher and the Sponsor are obliged to retain the data collected for the study for at least 5 years after its completion. Subsequently, the personal information will only be retained by the center for health care and by the promoter for other scientific research purposes if the patient has given consent to do so, and if permitted by applicable law and ethical requirements.

12. Handling of biological samples

No biological samples will be collected.

13. Finance and funding

Currently the project does not count with funding. We will use equipment that is already available for routine use at our center. The investigators will not receive any type of payment.

14. Publication

Data from this research will be made available to the scientific community in a timely manner regardless of the results. The investigators will comply with internationally agreed requirements for authorship and will approve any manuscripts prior to submission.
